# Timing and duration of bevacizumab treatment and survival in patients with recurrent ovarian, fallopian tube, and peritoneal cancer: a multi-institution study

**DOI:** 10.22514/ejgo.2023.002

**Published:** 2023-02-14

**Authors:** Talayeh S. Ghezelayagh, Emily S. Wu, Emma L. Barber, Minh D. Dao, Emese Zsiros, Renata R. Urban, Heidi J. Gray, Barbara A. Goff, Chirag A. Shah, Nikki L. Neubauer, James Y. Dai, Janos L. Tanyi, John B. Liao

**Affiliations:** 1 Division of Gynecologic Oncology, Department of Obstetrics and Gynecology, University of Washington, Seattle, WA 98195, USA; 2 Gynecologic Oncology Program, Department of Obstetics and Gynecology, University of New Mexico, Albuquerque, NM 87131, USA; 3 Division of Gynecologic Oncology, Department of Obstetrics and Gynecology, Northwestern University Feinberg School of Medicine, Robert H Lurie Comprehensive Cancer Center, Chicago, IL 60611, USA; 4 Jordan Center for Gynecologic Cancer at Penn, Perelman Center for Advanced Medicine, University of Pennsylvania Health System, Philadelphia, PA 19104, USA; 5 Department of Gynecologic Oncology, Roswell Park Comprehensive Cancer Center, Buffalo, NY 14263, USA; 6 Swedish Cancer Institute Gynecologic Oncology and Pelvic Surgery, Seattle, WA 98104, USA; 7 Gynecological Cancer Institute of Chicago, Oak Lawn, IL 60453, USA; 8 Public Health Sciences Division, Fred Hutchinson Cancer Center, Seattle, WA 98109, USA

**Keywords:** Ovarian cancer, Bevacizumab, Overall survival, Platinum sensitivity, Fallopian tube cancer

## Abstract

Bevacizumab has demonstrated significant benefit in recurrent ovarian, fallopian tube and peritoneal cancer (OC), but its optimal position within the sequence of systemic therapies remains controversial. Since rebound progression after bevacizumab has been observed in other cancers, and because bevacizumab is incorporated in several regimens used in the recurrent setting, the duration of treatment may impact survival. We sought to identify whether earlier bevacizumab exposure is associated with prolonged bevacizumab therapy and survival by conducting a multi-institution retrospective study of recurrent OC patients treated with bevacizumab from 2004–2014. Multivariate logistic regression identified factors associated with receiving more than six bevacizumab cycles. Overall survival by duration and ordinal sequence of bevacizumab therapy were evaluated using logrank testing and Cox regression. In total, 318 patients were identified. 89.1% had stage III or IV disease; 36% had primary platinum resistance; 40.5% received two or fewer prior chemotherapy regimens. Multivariate logistic regression demonstrated that primary platinum sensitivity (Odds Ratio (OR) 2.34, *p* = 0.001) or initiating bevacizumab at the first or second recurrence (OR 2.73, *p* < 0.001) were independently associated with receiving more than six cycles of bevacizumab. Receiving more cycles of bevacizumab was associated with improved overall survival whether measured from time of diagnosis (logrank *p* < 0.001), bevacizumab initiation (logrank *p* < 0.001), or bevacizumab discontinuation (logrank *p* = 0.017). Waiting one additional recurrence to initiate bevacizumab resulted in a 27% increased hazard of death (Hazard Ratio (HR) 1.27, *p* < 0.001) by multivariate analysis. In conclusion, patients with primary platinum sensitive disease who received fewer prior lines of chemotherapy were able to receive more cycles of bevacizumab, which was associated with improved overall survival. Survival worsened when bevacizumab was initiated later in the ordinal sequence of therapies.

## 1. Introduction

Despite numerous clinical trials in ovarian, fallopian tube, and peritoneal cancer, questions persist regarding the optimal use of bevacizumab, a monoclonal antibody targeting vascular endothelial growth factor (VEGF) [1, 2]. Exploratory analyses in major trials have described patient subgroups that may most benefit from bevacizumab, such as more advanced initial disease in ICON7, primary platinum sensitive recurrence in OCEANS or later platinum resistance in AURELIA [3–5]. Data in the primary and recurrent settings in ovarian cancer have also suggested better progression-free survival (PFS) with bevacizumab maintenance [6–8]. This data and the rebound effect on tumor growth observed in preclinical cancer models and in other cancers after discontinuation of anti-VEGF therapy have led to the hypothesis that survival benefits from bevacizumab may depend on maintenance therapy [9–14]. Furthermore, recent phase 3 data suggests that continuing bevacizumab beyond progression combined with chemotherapy may improve PFS in patients with recurrent ovarian cancer [15]. Prior to the advent of biologic agents such as bevacizumab, clinical factors including age, stage, performance status, tumor histology, and residual tumor volume were identified as independent prognostic factors for survival [16–18]. Whether these same prognostic factors retain equal significance with a biologic agent, such as VEGF-targeted therapy like bevacizumab, and what should guide how bevacizumab is used in recurrent ovarian cancer for the significant proportion of patients who do not receive it as part of initial therapy, remains unclear.

Bevacizumab has demonstrated great versatility in ovarian cancer, fallopian tube, and peritoneal cancer. It has been successfully deployed in frontline, maintenance, and recurrent disease. The role of bevacizumab in maintenance and the potential of an additive negative effect of rebound tumor growth with bevacizumab discontinuation led us to hypothesize that the timing and duration of bevacizumab use in the recurrent setting could impact survival. In the recurrent setting, bevacizumab has been successfully paired with several cytotoxic chemotherapies. Given the potential benefit from longer duration of bevacizumab therapy, and the many options for using bevacizumab reported in the recurrent setting, both alone and in several combinations, we undertook to study the timing of bevacizumab exposure and its impact on survival in recurrent ovarian, fallopian tube and peritoneal cancers.

## 2. Materials and methods

### 2.1 Patient selection

We performed a multi-institution retrospective analysis of patients who received bevacizumab for recurrent ovarian, fallopian tube, and peritoneal cancer from 2004 to 2014 at the following institutions: Northwestern University, Swedish Cancer Institute (Seattle), University of Pennsylvania, and University of Washington. Other criteria included having biopsy-confirmed diagnosis and the use of cytoreductive surgery and cytotoxic chemotherapy as part of their initial treatment. Patients were excluded if their medical records were incomplete regarding primary outcome, if they had a concurrent malignancy, or if they received bevacizumab as part of their initial treatment. The Institutional Review Board at each participating institution approved this study.

### 2.2 Data collection

Study variables, including demographic, clinicopathologic factors, treatment, and survival data were retrospectively abstracted from the medical record. Treatment variables included the number of cytotoxic chemotherapy regimens administered prior to initiation of bevacizumab, time from diagnosis to bevacizumab initiation, and the total number of bevacizumab cycles administered. The time from diagnosis to bevacizumab initiation was dichotomized as shorter than or equal to versus greater than the median duration for the entire population. Primary cytoreductive surgical outcome was categorized as optimal or suboptimal, where suboptimal surgery was defined as having greater than one centimeter of residual disease. Patients who progressed or experienced a recurrence within six months of completing primary adjuvant treatment containing a platinum agent were defined as having primary platinum resistant disease. Recurrence number at which bevacizumab therapy was initiated was treated initially as a dichotomous variable (recurrence number one or two versus three or greater) based on inclusion criteria used in prior randomized trials [19–21]. In secondary analyses, this variable was also treated continuously along the following scale: recurrence number 1, 2, 3, 4 or greater.

### 2.3 Statistical analysis

The primary outcome of interest was the total number of bevacizumab cycles administered across the patient’s lifetime. This was dichotomized as less than or equal to six cycles versus greater than six cycles. The dichotomization at six cycles was selected based on the median number cycles of chemotherapy and of bevacizumab administered in the OCEANS and AURELIA trials for recurrent ovarian cancer [5, 20]. The secondary outcome of interest was overall survival, which was measured both from the time of bevacizumab initiation and the time of bevacizumab discontinuation until date of death or last known contact. Patients still alive at the end of the study period were censored. Clinical and pathologic characteristics were compared with analysis of variance, Mann-Whitney, and Fisher’s exact tests.

For our primary analyses, logistic regression was used to identify independent factors associated with receiving greater than six cycles of bevacizumab. Significant factors identified through univariate analysis (Wald *p* < 0.05) were used in a multivariate model to identify independent associations. Sensitivity analysis was done with similar testing in a subgroup of patients that received bevacizumab after their first recurrence. Overall survival comparing receipt of greater than six and six or fewer bevacizumab cycles were then compared using logrank testing.

For our secondary analysis, logrank analysis was conducted examining overall survival stratified by recurrence number at which bevacizumab was initiated. A multivariate cox proportional hazards model was conducted to determine the increased risk of waiting one further recurrence before initiating bevacizumab. The model was adjusted with covariates based on *a priori* determinations from prior studies (age, stage, tumor histology, bevacizumab in combination with chemotherapy, primary platinum response, and time from diagnosis to bevacizumab initiation). Statistical significance was defined using the two-sided level of 0.05. STATA version 14 (StataCorp LLC, College Station, TX, USA) statistical software was used for all analyses.

## 3. Results

### 3.1 Characteristics of patients receiving bevacizumab

Complete information was available for 318 patients meeting inclusion criteria. Cohort demographics are outlined in Table 1. Mean age at initial diagnosis was 56.9 years. Median follow-up time in the study was 4.8 years. The majority of cancers were serous (77.7%), high grade (94.9%) and stage III–IV (89.1%) at diagnosis. Forty-one patients (12.9%) were initially treated with neoadjuvant therapy and 59 (18.6%) did not achieve optimal cytoreduction. Primary platinum resistance was noted in 115 (36.2%) patients. The median time from diagnosis to bevacizumab initiation was 33 months (range 3–403). Bevacizumab was combined with a cytotoxic agent in 277 (87%) of patients, most commonly cyclophosphamide (n = 151, 54.5%). Most were treated with bevacizumab at a dose of 5 or 10 mg/kg (n = 187, 59.2%), while the remainder received a dose of 15 mg/kg (n = 120, 40.8%). Fifty-eight (18.2%) patients were treated with bevacizumab at the time of their first recurrence, and 71 (22.3%) were treated with bevacizumab at the time of their second recurrence. At the end of the study, 293 patients had stopped bevacizumab. Reasons for discontinuation included toxicity (15.4%), progression or death (58.4%), treatment break or planned end of treatment (17.3%).

### 3.2 Factors associated with increased duration of bevacizumab therapy

Comparing patients who received greater than six cycles of bevacizumab with those who did not, there was no significant difference in mean age at diagnosis, histology, grade, stage, use of neoadjuvant chemotherapy, optimal cytoreduction, or time from diagnosis to bevacizumab initiation (all *p* > 0.05) (Table 1). There was also no difference in the rate of patients stopping bevacizumab due to toxicity between the two groups (*p* = 0.209). Patients who received greater that six cycles of bevacizumab were less likely to have primary platinum resistance (*p* = 0.003), to receive bevacizumab in combination with chemotherapy (*p* = 0.027), and have received a starting bevacizumab dose of 15 mg/M^2^ (*p* < 0.001). A significantly increased proportion of patients who received greater than six cycle of bevacizumab initiated bevacizumab treatment after their first or second recurrence (*p* < 0.001).

**FIGURE 1.**
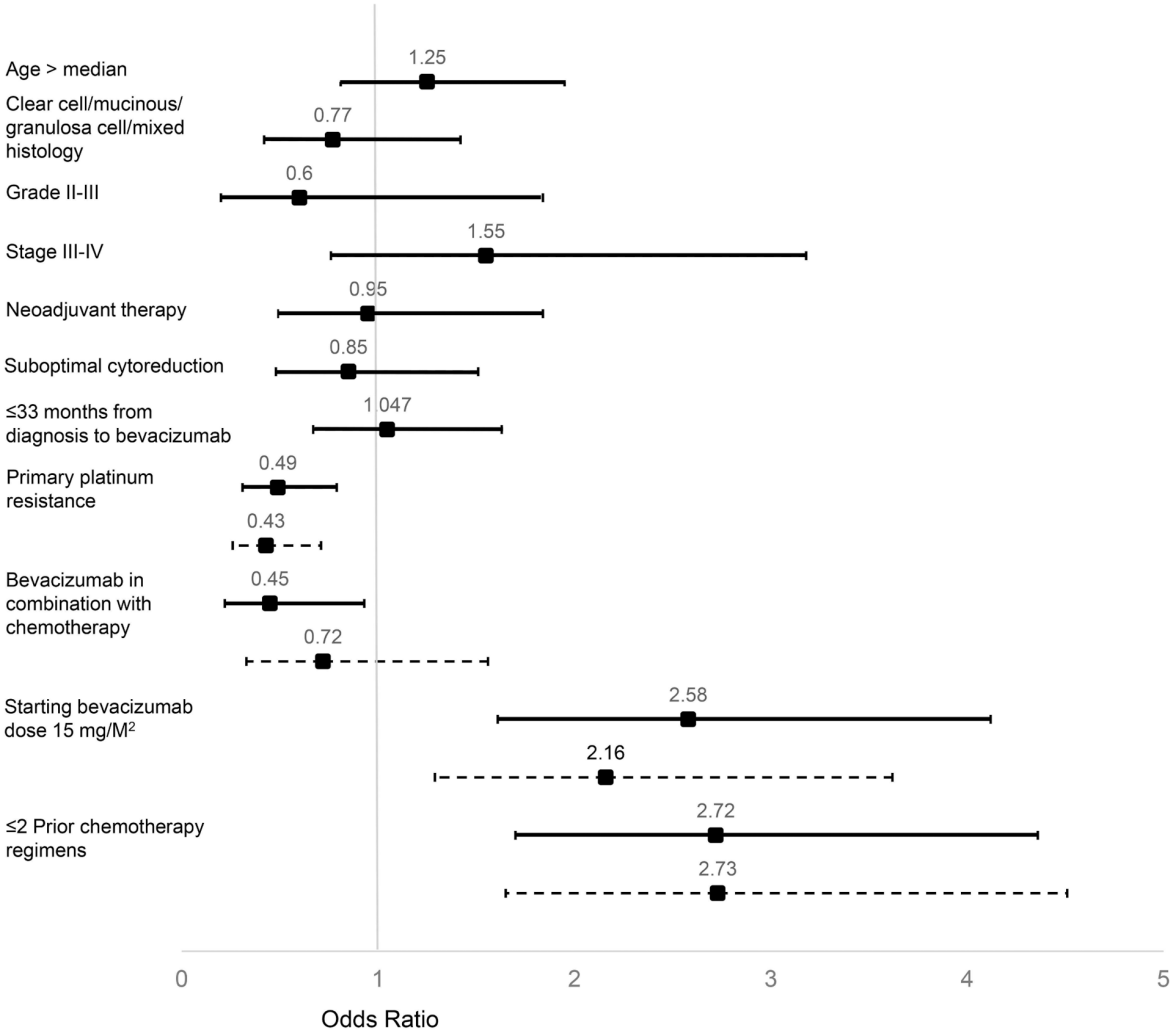
Factors associated with receiving greater than six cycles of bevacizumab in patients treated for recurrent ovarian, fallopian tube, or peritoneal cancer, based on logistic regression. Odds ratio with 95% confidence intervals are displayed. Factors significant on univariate analysis (solid line) were combined into a multivariate model (dashed line).

On univariate logistic regression analysis of clinicopathologic factors, primary platinum resistance (OR 0.49, *p* = 0.003) and receipt of bevacizumab in combination with chemotherapy (OR 0.45, *p* = 0.030) were significantly associated with receiving six or fewer cycles of bevacizumab (Fig. 1). Age, tumor histology, stage, neoadjuvant therapy, suboptimal cytoreduction, and time from diagnosis to bevacizumab initiation were not significantly associated with duration of bevacizumab treatment. Receiving bevacizumab after the first or second recurrence (OR 2.72, *p* < 0.001) and receiving starting bevacizumab dose of 15 mg/M^2^ (OR 2.58, *p* < 0.001) was significantly associated with receiving greater than six cycles of bevacizumab. Time from diagnosis to initiation of bevacizumab was not found to be associated with length of bevacizumab therapy (OR 1.047, *p* = 0.838).

Factors significant in univariate analysis were combined into a multivariate model (Table 2). In multivariate analysis, primary platinum resistance was independently associated with a 57% lesser odds of receiving greater than six cycles of bevacizumab (OR 0.43, *p* = 0.001; OR 2.34 for primary platinum sensitivity). Multivariate analysis also showed that receiving a starting dose of bevacizumab of 15 mg/M^2^ bevacizumab or receiving bevacizumab after only the first or second recurrence was independently associated with an increased odds of receiving greater than six cycles of bevacizumab (OR 2.16, *p* = 0.003 and OR 2.73, *p* < 0.001, respectively).

In our sensitivity analysis, similar logistic regression was done for the subset of patients who received bevacizumab on their first recurrence. In the multivariable model, primary platinum resistance (OR 0.139, *p* = 0.007) and neoadjuvant therapy (OR 0.135, *p* = 0.019) were independently associated with receiving six or fewer cycles of bevacizumab.

### 3.3 Duration of bevacizumab therapy and effect on overall survival

**FIGURE 2.**
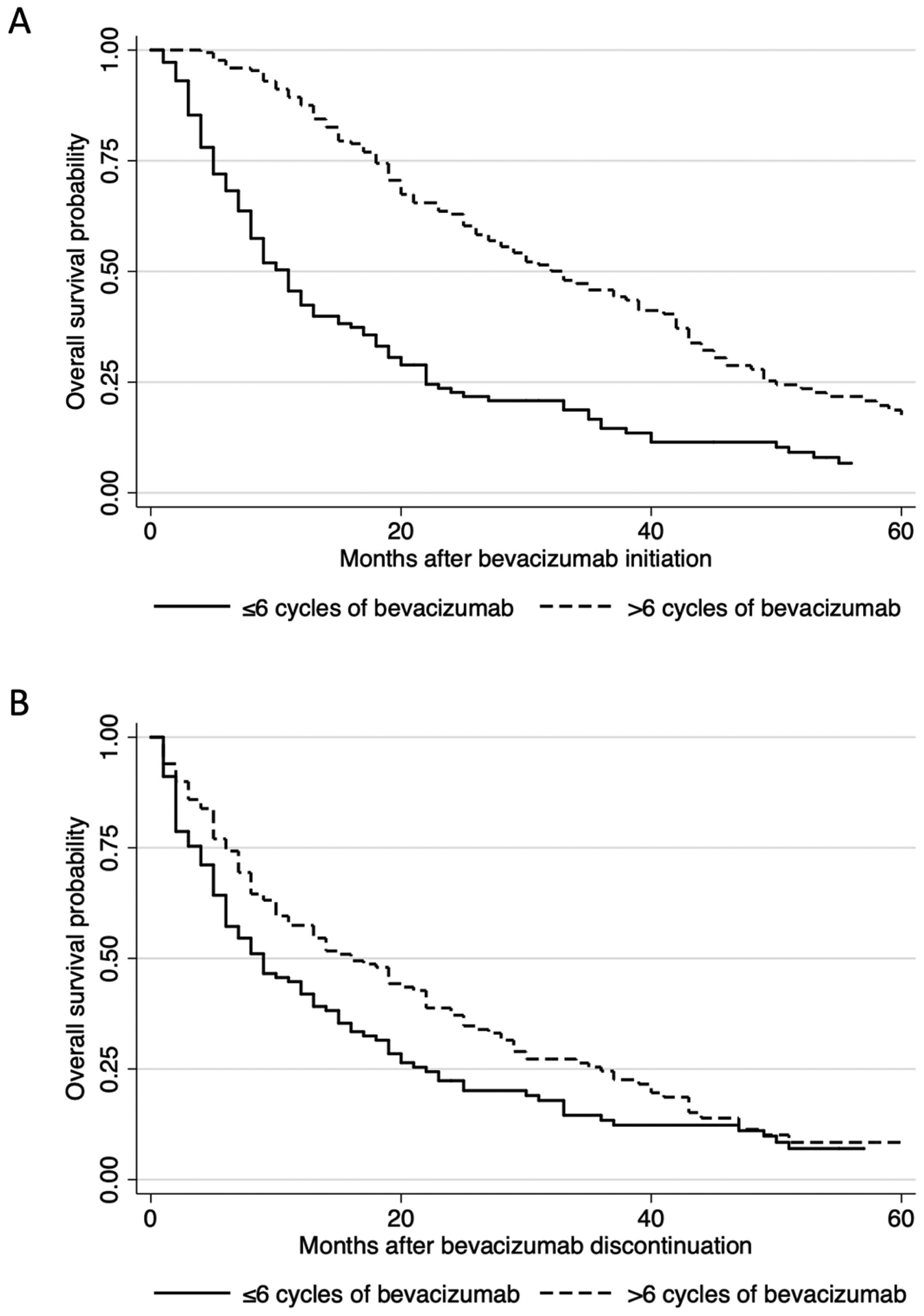
Kaplan-Meier plots of overall survival stratified by number of bevacizumab cycles received, measured from (A) the time of bevacizumab initiation (median 33 *vs*. 11 months, logrank *p* < 0.001) and (B) the time of bevacizumab discontinuation (median 16 *vs*. 9 months, logrank *p* = 0.017).

Receiving more cycles of bevacizumab for recurrence was associated with increased overall survival from time of diagnosis (hazard ratio 0.62, *p* < 0.001; logrank *p* < 0.001). This relationship persisted when measuring survival from both bevacizumab initiation (hazard ratio 0.40, *p* < 0.001) and when measuring from bevacizumab discontinuation (hazard ratio 0.73, *p* = 0.020). The median overall survival after bevacizumab initiation was 33 months in those who received greater than six cycles of bevacizumab and 11 months in those who received six or fewer, with a persistent significant difference in survival curves (logrank *p <* 0.001, Fig. 2A). On sensitivity analysis limited to patients receiving only bevacizumab without concomitant chemotherapy, increased cycles of bevacizumab is still associated with increased overall survival (hazard ratio 0.26, *p* = 0.014, logrank *p* = 0.008). This suggests that independent of the added chemotherapy, there is a benefit of longer duration of bevacizumab therapy on survival.

When survival time is measured from bevacizumab discontinuation, the curves remain persistently different, with no convergence until 40 months (logrank *p* = 0.017, Fig. 2B). Median survival from bevacizumab discontinuation was 16 months and 9 months for those who received greater than six cycles of bevacizumab compared to those who received six or fewer. Stratifying by starting dose of bevacizumab did not affect survival patterns.

### 3.4 Timing of bevacizumab therapy

**FIGURE 3.**
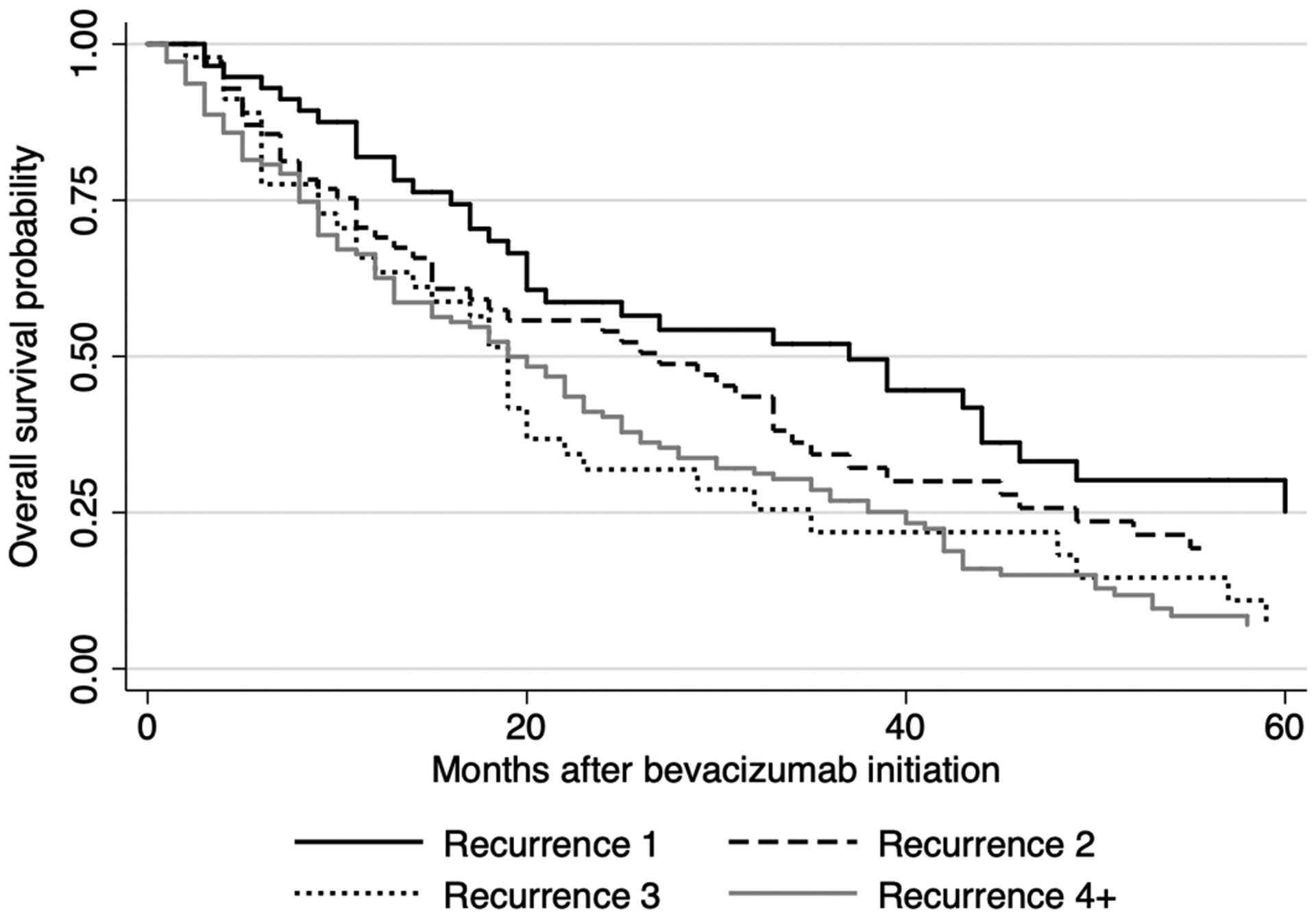
Kaplan-Meier plot of overall survival measured from time of bevacizumab initiation, stratified by recurrence number at time of bevacizumab initiation. Median overall survival 37 months after recurrence 1, 27 months after recurrence 2, 19 months after recurrence 3, and 19 months after 4 or more recurrences (logrank *p* = 0.0046).

Overall survival measured from bevacizumab initiation diverged depending on recurrence number at which bevacizumab therapy was initiated (Fig. 3, logrank *p* = 0.0046). Patients receiving bevacizumab only after the third or fourth recurrence tended to have worse survival throughout the follow-up period (median survival after bevacizumab initiation 37, 27, 19, and 19 months if initiated at the first, second, third, or fourth recurrence, respectively). On multivariate Cox Proportional Hazards analysis, after adjusting for *a priori* defined factors (age, stage, tumor histology, bevacizumab in combination with chemotherapy, primary platinum response, and time from diagnosis to bevacizumab initiation), waiting one additional recurrence cycle to add bevacizumab resulted in a 27% increased hazard of death (HR 1.27, *p* < 0.001).

## 4. Discussion

In our cohort of heavily pre-treated patients with recurrent ovarian, fallopian tube, and peritoneal cancer, we found that initiating bevacizumab earlier in a patient’s sequence of recurrences is independently associated with the response, measured both by duration of bevacizumab therapy and overall survival. Patient who were able to receive more cycles of bevacizumab were subsequently confirmed to have improved overall survival, in contrast to historically used cytotoxic chemotherapies where more cycles have not always been associated with improved survival [22–24]. This association remained even when focusing on patients who received bevacizumab monotherapy. Duration of bevacizumab is a surrogate of response to therapy in recurrence ovarian cancer, as additional cycles are traditionally given as maintenance therapy in patients with stable or improving disease burden. Our study however further explored whether the timing of bevacizumab therapy makes a difference on patient outcomes.

Phase III trials have shown improved progression-free survival with bevacizumab in both primary treatment [3, 7] and in treating recurrence [5, 8, 20]. This has raised questions regarding the optimal timing of bevacizumab therapy and whether it is best used as adjuvant chemotherapy or if it is better “reserved” for recurrence [1, 2]. If the latter, at what point in the trajectory of recurrence, persistence, and progression should bevacizumab be initiated has not been established [2]. It is difficult to derive insight regarding this question from the phase III trials that have been published to date, since there is data for use in both the platinum sensitive and platinum resistant settings. The OCEANS trial, which studied bevacizumab use in recurrent platinum-sensitive ovarian cancer, excluded participants who had received any prior chemotherapy in the recurrent setting [5]. The MITO16b/MANGO-OV2/ENGOTOV17 trial saw a three month improvement in progression free survival when bevacizumab was given in combination with a platinum doublet beyond progression in patients with platinum sensitive ovarian cancer [15]. The AURELIA trial, which studied bevacizumab in recurrent platinum-resistant ovarian cancer, only included patients who had up to two prior anticancer regimens, did not include patients previously treated with bevacizumab, and did not stratify their results by number of prior anticancer regimens [20].

Our study suggests that introducing bevacizumab earlier in a patient’s sequence of treatment regimens confers additional benefit. This may allow patients to benefit earlier from VEGF tumor suppression, which preclinical data has suggested is necessary to maintain tumor control [25]. This concept may lend further justification for using maintenance bevacizumab to sustain response in the recurrent setting. Even after progression on bevacizumab, a prior phase II study has suggested that adding oral cyclophosphamide at the time of progression may result in at least stable disease within a subset of patients, potentially due to anti-angiogenic activity attributed to cyclophosphamide [26]. Metronomic cyclophosphamide is thought to suppress tumor growth by inhibiting angiogenesis, and has demonstrated activity when administered in combination with bevacizumab [21, 27]. Continuing bevacizumab after progression is now being studied in other tumors, including colorectal and breast cancer [28, 29].

Data in the primary and recurrent setting in ovarian cancer suggest better PFS when bevacizumab maintenance is administered [6–8]. The concept that benefit from bevacizumab may depend on ongoing use has also been introduced in other cancers, where rebound tumor progression after discontinuing bevacizumab has been hypothesized [10, 13, 14]. A rebound effect on tumor growth has been observed in preclinical cancer models and in other cancers after discontinuation of anti-VEGF therapy [9–14]. However, in our study, survival curves did not reflect rebound tumor progression after stopping bevacizumab, converging only after 40 months. It is possible that in ovarian cancer, the benefits of bevacizumab extend beyond the duration of treatment or the number of bevacizumab cycles received may be a surrogate for more favorable tumor biology.

Although other retrospective studies have analyzed factors associated with survival in heavily pretreated patients who received bevacizumab in recurrent ovarian cancer, our study looked at duration of bevacizumab therapy, since bevacizumab can be combined with a number of cytotoxic chemotherapies, and to assess how that relates to improved survival [30, 31]. In a previously reported study, additional prior chemotherapy regimens before bevacizumab exposure was reported to be associated with longer overall survival, although that study included patients receiving bevacizumab as part of their front-line treatment and may have been influenced by bias from patients with a more indolent disease course [31]. Treatment-free interval (TFI) was also reported to be a significant prognostic factor, but can also be a surrogate for more favorable tumor biology [31]. When patients who received up-front treatment with bevacizumab were excluded in a different retrospective analysis, there was an improvement in overall survival when bevacizumab was combined with other cytotoxic agents, but an association with number of prior regimens was not found [30]. That study focused on an entirely platinum-resistant cohort, however, so represents a slightly different population from the patients presented here.

Our study is limited by its retrospective design and heterogeneity in the cohort, such as variability in treatment regimens across institutions and providers and variation in the number of times across a patient’s treatment course bevacizumab was used. While performance status has been shown to be prognostic for survival, we were unable to account for that due to limitations in the medical record review. We were only able to account for primary platinum response, rather than platinum response at the time of initiating bevacizumab, although our sensitivity analysis showed similar conclusions when limited to patients at the time of their initial recurrence. We could not control for post-bevacizumab therapy, which may influence the assessment of overall survival. However, overall survival is still considered the most objective and accepted endpoint in ovarian cancer trials due to its relative freedom from bias [32]—particularly in a retrospective study where assessments in disease status is not standardized across patients. OCEANS and AURELIA trials for bevacizumab combinations in platinum sensitive and recurrent disease were reported during the the last year that patients were included in this study, so those regimens are underepresented in our cohort [4, 5]. While recurrent platinum resistant and recurrent platinum sensistive patients were both included in this study, contributing to the heterogeneity of the study cohort, the continued clinical utility of the platinum sensitive/resistant paradigm has also been questioned [33]. Lastly, as a retrospective study, despite adjustments made in our survival analyses to show association between duration of bevacizumab and survival, we cannot fully account for potential selection bias as clinical features may also favor more sensitive tumor biologies.

Strengths of this study include large sample size, duration of follow-up, multi-institutional source of data, and survival analyses from bevacizumab initiation and discontinuation. The four institutions from which the study population is drawn represent a diversity of geographic areas and practice patterns. Although this leads to heterogeneity in our study population, it may also reflect greater generalizability. By conducting analyses in which survival time is defined as time from bevacizumab initiation and as time from bevacizumab discontinuation, we were able to better parse out potential confounding factors such as immortal time bias.

Our data correlating more cycles of bevacizumab with improved overall survival is at odds with what was reported in final survival analysis for Gynecologic Oncology Group study 218, which studied the use of bevacizumab in frontline therapy [34]. The use of bevacizumab in primary treatment is not universal, and our study included only patients that did not receive bevacizumab as part of initial treatment. Based on studies of neoadjuvant chemotherapy, frontline cytotoxic chemotherapy regimens can significantly alter the tumor microenvironment and so may alter response to subsequent biologic therapies such as bevacizumab [35]. This would be consistent with a metaanalysis showing improved overall survival when bevacizumab is used in the recurrent setting compared to no significant advantage in frontline [8].

## 5. Conclusions

On adjusted analysis, patients with recurrent ovarian cancer with two or fewer prior lines of cytotoxic chemotherapy, higher starting dose of bevacizumab, or with primary platinum sensitive disease were more likely to receive greater than six bevacizumab cycles. Patients who received greater than six cycles of bevacizumab also experienced improved overall survival measured from time of diagnosis, initation of bevacizumab and discontinuation of bevacizumab. This study highlights considerations for optimizing the use of bevacizumab and may assist in further defining the subgroup of patients that will derive the most benefit. In addition to survival, the impact of bevacizumab on toxicity, quality of life and cost must also be considered. There may be an optimal window for bevacizumab in the ordinal sequence of ovarian cancer recurrences that would justify future prospective trials to study strategies employing sequential bevacizumab combinations to extend the anti-angiogenic component of tumor control.

## Figures and Tables

**TABLE 1.** Patient demographic, clinical and treatment characteristics.

	Total Cohort (n = 318)*	≤6 cycles of bevacizumab (n = 144)*	>6 cycles of bevacizumab (n = 174)*	*p* value^†^
Mean age at diagnosis (standard deviation)	56.9 (11.2)	56.4 (11.9)	57.3 (10.6)	0.491^‡^
Histology				
Serous	244 (77.7%)	104 (72.7%)	140 (81.8%)	0.253
Endometrioid	21 (6.7%)	14 (9.8%)	7 (4.1%)	
Clear Cell	13 (4.1%)	8 (5.6%)	5 (2.9%)	
Mucinous	4 (1.3%)	2 (1.4%)	2 (1.2%)	
Granulosa cell	3 (1.0%)	1 (0.7%)	2 (1.2%)	
Mixed	29 (9.2%)	14 (9.8%)	15 (8.8%)	
Grade				
I	14 (5.1%)	5 (3.8%)	9 (6.2%)	0.421
II–III	262 (94.9%)	126 (96.2%)	136 (93.8%)	
AJCC/FIGO Stage^§§^				
I	19 (6.1%)	12 (8.3%)	7 (4.2%)	0.496
II	15 (4.8%)	7 (4.9%)	8 (4.7%)	
III	236 (75.6%)	107 (74.3%)	129 (76.8%)	
IV	42 (13.5%)	18 (12.5%)	24 (14.3%)	
Neoadjuvant therapy	41 (12.9%)	19 (13.3%)	22 (12.7%)	1.000
Suboptimal surgery	59 (18.6%)	29 (20.4%)	30 (18.3%)	0.665
Primary platinum resistance	115 (36.2%)	65 (45.1%)	50 (28.9%)	0.003
Bevacizumab combined with chemotherapy	277 (87.0%)	132 (91.7%)	145 (83.3%)	0.027
Starting Bevacizumab dose				
5–10 mg/M^2^	187 (59.2%)	102 (71.3%)	85 (49.1%)	<0.001
15 mg/M^2^	129 (40.8%)	41 (28.7%)	88 (50.9%)	
Median recurrence number when bevacizumab initiated (range)	3 (1–16)	4 (1–16)	2 (1–16)	<0.001^§^
Bevacizumab initiated on recurrence number:				
1	58 (18.2%)	16 (11.1%)	42 (24.1%)	<0.001
2	71 (22.3%)	24 (16.7%)	47 (27.0%)	
3	47 (14.8%)	18 (12.5%)	29 (16.7%)	
4+	142 (44.7%)	86 (59.7%)	56 (32.2%)	
Median months from diagnosis to bevacizumab initiation (standard deviation)	33 (36.5)	33.5 (32.3)	33 (39.8)	0.926^§^
≤33 months from diagnosis until bevacizumab initiation	161 (50.6%)	72 (50.0%)	89 (51.2%)	0.464

* Cells of columns 2–4 are in n(%) format unless otherwise described.

† Comparison testing done between those that received ≤6 and >6 cycles of bevacizumab, with Fisher’s Exact test used unless otherwise indicated.

‡ Two sample t-test.

§ Mann-Whitney test.

§§ American Joint Committee on Cancer (AJCC) and International Federation of Gynecology and Obstetrics (FIGO)

**TABLE 2.** Factors associated with >6 cycles of bevacizumab given in patients treated for recurrent ovarian, fallopian tube, or peritoneal cancer.

	Univariate odds ratio, CI* (*p*)	Multivariate odds ratio, CI* (*p*)
Age > median	1.25, 0.81–1.95 (0.316)	–
Clear cell/mucinous/granulosa cell/mixed histology (*vs*. serous/endometrioid)	0.77, 0.42–1.42, (0.403)	–
Stage III–IV	1.55, 0.76–3.18 (0.231)	–
Neoadjuvant therapy	0.95, 0.49–1.84 (0.881)	–
Suboptimal surgery	0.85, 0.48–1.51 (0.584)	–
Primary platinum resistance	0.49, 0.31–0.79 (0.003)	0.43, 0.26–0.7 (0.001)
BEV^†^ with combination chemotherapy	0.45, 0.22–0.93 (0.030)	0.72, 0.33–1.56 (0.401)
Starting dose of bevacizumab 15 mg/M^2^	2.58, 1.61–4.12 (<0.001)	2.16, 1.29–3.633 (0.003)
≤2 prior chemo regimens before BEV^†^	2.72, 1.70–4.36 (<0.001)	2.73, 1.65–4.51 (<0.001)

* Confidence Interval (CI).

† Bevacizumab (BEV).

## Data Availability

The datasets used and analyzed during the current study are available from the corresponding author on reasonable request.
